# Efficient identification of mental health problems in refugees in Germany: the Refugee Health Screener

**DOI:** 10.1080/20008198.2017.1389205

**Published:** 2017-11-07

**Authors:** Elisa Kaltenbach, Eva Härdtner, Katharin Hermenau, Maggie Schauer, Thomas Elbert

**Affiliations:** ^a^ Department of Psychology, University of Konstanz, Konstanz, Germany

**Keywords:** Refugee, asylum seeker, screening, mental health, psychiatric disorder, psychometric, validation, Europe, Germany, refugiado, solicitante de asilo, prueba de detección, salud mental, trastorno psiquiátrico, psicométrico, validación, Europa, Alemania, • The Refugee Health Screener (RHS) showed a good feasibility, reliability, and validity as a screening instrument within a heterogeneous refugee sample in Germany., • The RHS detects common mental health problems such as PTSD, depression, anxiety, or somatization problems., • The RHS is valid as a self-rating and interview., • A shorter version of the RHS, the RHS-13, is more time-efficient and equally valid.

## Abstract

**Background:** A substantial number of refugees present with mental disorders. This appears particularly acute in the currently increasing refugee populations in Europe. Although EU guidelines demand the identification and support of vulnerable individuals such as survivors of trauma, no adequately validated and comprehensive mental health screening instruments for refugees residing in Europe currently exist.

**Objective:** We studied the feasibility, validity, and reliability of the Refugee Health Screener-15 (RHS-15) – a time-efficient and easy-to-implement screening developed by Hollifield et al. (2013) – as a self-rating and interview instrument.

**Methods:** A sample of refugees from different countries (*N* = 86), representative of those who had arrived around the turn of the year 2015/2016 in Germany, filled in the RHS-15 on their own. A semi-structured clinical interview was later conducted with a random subsample (*n* = 56).

**Results:** Fifty-two percent of the refugees examined screened positive in the RHS-15, thus indicating current mental health problems. The RHS-15 showed a good feasibility, reliability, and validity in both the self-rating and the interview version. It detected clinically relevant mental health problems when PTSD, depression, anxiety, or somatization problems were present. A shorter 13-item version proved to be equally valid.

**Conclusions:** Together with previous research on the RHS in refugees living in the US, this suggests that the RHS is a time-efficient and accurate instrument that is able to detect common mental health problems in a wide range of refugees. Prospectively, the RHS could be used as an instrument for identifying vulnerable refugees, for example, by integrating it in the initial medical examination in the host community, thereby initiating support.

## Abstract (es)

**Planteamiento:** Un número considerable de refugiados presenta trastornos mentales. Esto parece ser particularmente grave en poblaciones de refugiados que actualmente están aumentando en Europa. Aunque las directrices de la UE exigen identificar y apoyar a individuos vulnerables, como los sobrevivientes de trauma, actualmente no existen instrumentos de detección de salud mental adecuados e integrales para los refugiados que residen en Europa.

**Objetivo:** Se estudió la viabilidad, la validez y la fiabilidad de la Pruebas de salud para refugiados - 15 (RHS-15, siglas en inglés de Refugee Health Screener) - una evaluación eficiente y fácil de implementar, desarrollada por Hollifield et al. (2013) - como instrumento de autoevaluación y entrevista.

**Métodos:** Una muestra de refugiados de diferentes países (N = 86) –representativa de aquellos que habían llegado a Alemania a finales de 2015 y principios de 2016– rellenó el RHS-15 por su cuenta. Posteriormente se realizó una entrevista clínica semiestructurada con una submuestra aleatoria (n = 56).

**Resultados:** El 52% de los refugiados examinados obtuvieron resultados positivos en la RHS-15, lo que indicaba que tenían problemas de salud mental en la actualidad. La RHS-15 mostró una buena viabilidad, fiabilidad y validez tanto en la autoevaluación como en la versión de la entrevista. Detectaba problemas de salud mental clínicamente relevantes cuando había TEPT, depresión, ansiedad o somatización. Una versión más corta de 13 ítems resultó ser igualmente válida.

**Conclusiones:** Junto con investigaciones previas sobre la RHS en refugiados que viven en los Estados Unidos, esto sugiere que la RHS es un instrumento eficiente y preciso, capaz de detectar problemas de salud mental comunes en una amplia gama de refugiados. Posiblemente, la RHS podría ser utilizado como instrumento para identificar a refugiados vulnerables, por ejemplo, integrándolo en el examen médico inicial en la comunidad de acogida, con lo cual ya se comenzaría a apoyarles.

## Abstract (zh)

**背景：**显著数量的难民表现出精神障碍。这一现象在现在欧洲数量激增的难民中尤为突出。尽管欧盟指导方针需要对弱势个体进行识别和支持，比如创伤的生还者，但是在欧洲目前还没有经过验证的综合性的心理健康筛查工具。

**目标：**我们研究了难民健康筛查工具-15（RHS-15）作用自评和访谈工具的可行性和信效度，这是由Hollifield等人在2013年开发的一种节约时间的且容易使用的工具。

**方法：**从不同国家筛选出的难民（N=86)，代表了2015/2016年到达德国的难民。他们自行填写了RHS-15，其中一个随机子样本（n=56）又接受了半结构化临床访谈。

**结果：**结果：52%的难民在RHS-15测试中筛选出阳性症状，这表明他们现有心理健康问题。RHS-15在自评和访谈的版本都表现出很好的可行性和信效度。它探查了当PTSD、抑郁，焦虑或躯体化问题出现时临床上会出现心理健康问题。一个13题的简版也同样有效。

**结论：**与之前对美国难民进行的RHS研究一同表明， RHS是一个节约时间的、准确的测量工具， 它能够在大量难民中检测出常见的心理健康问题。有可能的话， RHS能够作为工具用来识别弱势难民。比如在接受难民的社区中和初级医疗检查一起使用， 然后开始提供支持。

## 1. Introduction

War, persecution, and violence have led to record-high numbers of people being forcibly displaced, with figures estimated at 65.6 million people in 2016 (United Nations High Commissioner for Refugees [UNHCR], 2017). In Europe, the number of first-time asylum applications increased strongly in 2015 and 2016, with Germany being the largest recipient (Eurostat, 2017). The majority of refugees came from current or former war populations such as Syria, Afghanistan, and Iraq (Eurostat, 2017). Mental health problems are overrepresented in refugee as compared to non-refugee populations (Porter & Haslam, 2005; Priebe, Giacco, & El-Nagib, 2016). Given the refugees’ heterogeneity and differences in pre-flight, flight, and postmigrational experiences, it is unsurprising that reviews have found a high intersurvey variability (e.g. Miller, Elbert, & Rockstroh, 2005; Steel et al., 2009). However, prevalence rates of 30% for posttraumatic stress disorder (PTSD) and depression are a typical estimate (Miller et al., 2005; Steel et al., 2009). Studies examining the mental health of the present groups of refugees report comparable rates (e.g. Alpak et al., 2015; Führer, Eichner, & Stang, 2016). The ever-growing number of refugees with traumatic experiences (Alpak et al., 2015; UNHCR, 2017) and the associated high rates of mental health problems have been termed a ‘mental health crisis’ (Schauer, 2016). Hence, there is a strong imperative to assess mental health needs – for example, by integrating mental health screening into the initial medical examination already in place in many countries (e.g. Elbert, Wilker, Schauer, & Neuner, 2016; Rhema, Gray, Verbillis-Kolp, Farmer, & Hollifield, 2014).

For EU states, this is underlined by the directive of the European Parliament and of the Council (directive 2013/33/EU) stating that EU states have to assess refugees’ special needs within a reasonable time period and accordingly address these needs in providing medical and psychological treatment, especially for vulnerable persons. This includes, amongst others, minors, victims of severe violence or human rights violations, and those suffering from mental illnesses.

Given the substantial rates of mental illness in refugees, the high use of primary health care and hospitalization rates is not surprising – however, access to mental health services is very low (Bell & Zech, 2009; Hadgkiss & Renzaho, 2014). This is partly due to legal restrictions in the use of health services and barriers such as communication problems, poor health literacy, limited available care, and stigmatization associated with the use of mental health services (Kluge et al., 2012; Norredam, Mygind, & Krasnik, 2005). However, a study by Bozorgmehr and Razum (2015) shows that restricted access to health care is connected with higher health care costs. Further studies found that receiving appropriate and timely treatment is connected with enduring improvements in the refugees’ mental health and a lower use of emergency care (Lamkaddem et al., 2014; Song, Kaplan, Tol, Subica, & de Jong, 2015). Respectively, scientists and non-governmental organizations recommend the inclusion of preventive care and assistance for asylum seekers with mental health issues (e.g. Führer et al., 2016; Katsapaou, 2013). With this goal in mind, state-wide programmes in the USA have included mental health screening in the initial medical examination. They found good results in the feasibility and acceptability of mental health screenings in the public health system (Hollifield et al., 2016; Savin, Seymour, Littleford, Bettridge, & Giese, 2005). Potential barriers in screening and acceptance of referral to mental health services include problems in communication, confidentiality, literacy, stigma, limited care capacities, and difficulties in contacting the refugees (e.g. Al-Obaidi, West, Fox, & Savin, 2015).

In spite of existing research indicating the feasibility and usefulness of mental health screening, most short screening instruments only test a limited range of mental problems, or are either insufficiently validated or not validated at all (Hollifield et al., 2013, 2002). Despite the directive 2013/33/EU, screening instruments validated for refugees who have fled to Europe are scarce (Söndergaard, Ekblad, & Theorell, 2003). Furthermore, we found no studies specifically examining whether a mental health screening can be self-administered, thereby requiring less resources for wide-scale implementation.

One instrument that fulfils some of these issues is the Refugee Health Screener-15 (RHS-15; Hollifield et al., 2013). The RHS-15 screens for multiple mental problems, shows good psychometric properties, and is feasible in public health settings (Hollifield et al., 2016, 2013; Johnson-Agbakwu, Allen, Nizigiyimana, Ramirez, & Hollifield, 2014; Polcher & Calloway, 2016). It assesses symptoms of the most common mental problems in refugees: PTSD, depression, and anxiety symptoms. It can be administered as an interview or a self-rating and is available in several languages. Studies have found a good reliability and concurrent and predictive validity for refugees from Bhutan, Burma, and Iraq staying in the US (Hollifield et al., 2016, 2013). They identified a one factor structure for the questionnaire. A recent study by Hollifield et al. (2016) introduced a 13-item version, excluding items 14 and 15, with potential for being more efficient.

We therefore evaluated the feasibility, reliability, validity, and the mode of implementation of the RHS-15, and its shorter version the RHS-13, for refugees who have come to Germany. The study was conducted in Germany because its refugee characteristics are comparable to those of other EU countries (Bundesamt für Migration und Flüchtlinge [Federal Office for Migration and Refugees], 2017; Führer et al., 2016; Gäbel, Ruf, Schauer, Odenwald, & Neuner, 2006).

## 2. Method

### 2.1. Sample

The participating refugees lived in a refugee accommodation in a rural area in southern Germany. Of the 89 people (> 12 years) living in this accommodation, 97% (*N* = 86 of 89) participated. The average age of the total sample was *M* = 28.76 (*SD* = 11.23, range 12.08–65.83, *N* = 86) and 64% (*n *= 55 of 86) were male. The majority came from Syria (58%), followed by Afghanistan (9%), Albania (8%), Kosovo (7%), Serbia (7%), Iraq (4%), Macedonia, Somalia, and Georgia (each 2%). The length of stay in Germany was *M* = 6.53 months (*SD* = 2.99, range 3–24, *N* = 86). Participants reported an average of *M* = 10.44 years (*SD* = 4.01, range 0–20, *N* = 86) of formal education, 7% (*n* = 6) were illiterate.

### 2.2. Study design

**Figure 1.**
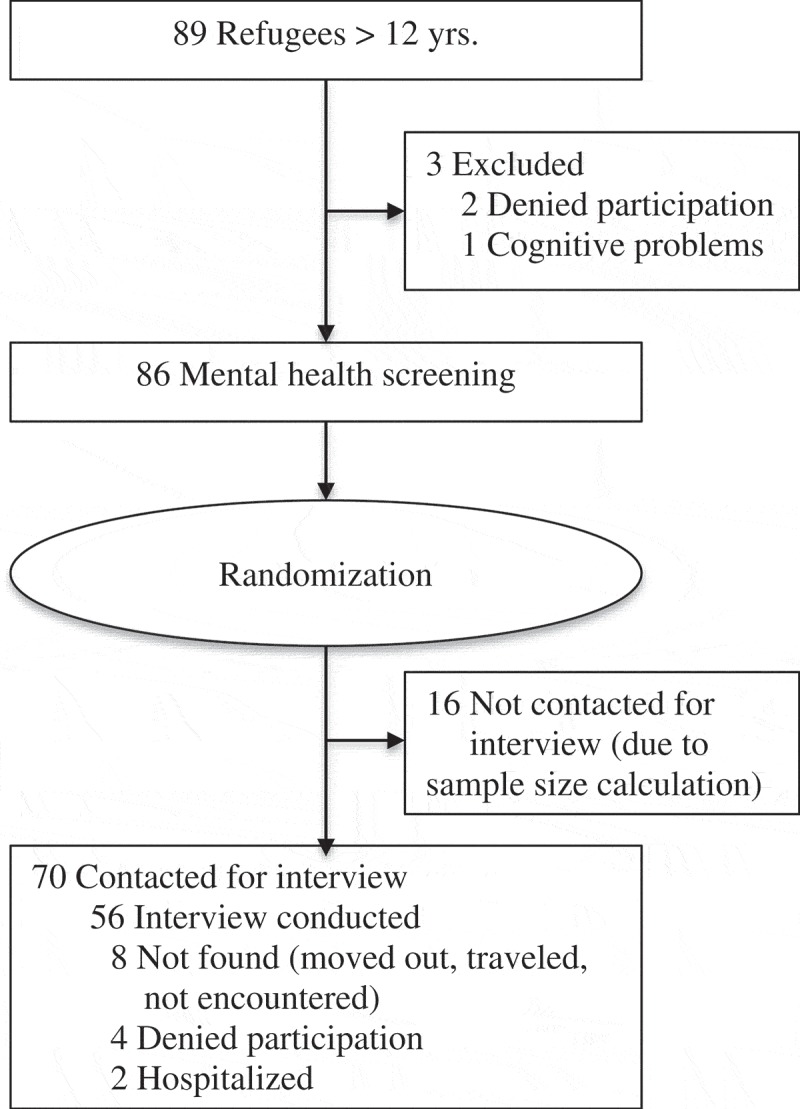
Flow of participants.

The study consisted of two consecutive parts: (1) Screening: Respondents completed the RHS-15 as a self-rating questionnaire in their respective first language. In cases of illiteracy, an interpreter read the questions word-for-word to the participant. Screenings took 10–30 minutes. (2) Semi-structured clinical interview: A randomized subsample was selected for semi-structured clinical interviews. The interviews were conducted approximately 1–2 weeks after the screening and took 1.5 h on average. Following Terwee et al. (2007), we decided that a minimum of 50 interviews should be conducted. We expected a drop-out rate of 20–30% because of experiences in previous studies. We first generated a randomized sequence of all participants and selected the first 70 persons in this sequence for the interviews. Out of this group, 80% (*n* = 56) participated in the interview. For further information on the study design, see Figure 1. No significant differences could be detected between the participants who only participated in the screening and those who additionally participated in the validation interview (see Supplemental data File 1).

### 2.3. Sampling procedures

The refugees were originally randomly referred to the particular accommodation centre by government authorities. All refugees above the age of 12 years (*N* = 89) were invited to participate in the study. To keep the sample as representative and realistic as possible, the only exclusion criterion was suffering from severe cognitive deficits (no conversation possible).

Refugees were informed of the study and its purpose in a general house meeting, with posters, and by knocking on every door and personally informing the residents. Screenings and interviews began with a comprehensive explanation of the study and a written informed consent for the participant. For minors, the consent of the legal guardian was also obtained. Participants were assured that participation was voluntary, that all data collected would be confidential, and that no monetary compensation would be offered. In case of a positive screening, treatment options were discussed with the participant and, if desired, participants were referred to appropriate services. The Ethical Review Board of the University of Konstanz approved the study.

### 2.4. Setting

Data collection was completed over three weeks in 2016. Screenings and interviews were conducted by clinical psychologists (*N* = 13) trained and experienced in the work with refugees and the detection of mental health problems. Interpreters (*N* = 9; Albanian, Arabic, Farsi, Kurdish, Russian, Serbian) who were trained in translating in the mental health context and who had extensive experience in translating were present throughout the study. The psychologists conducting the validation interviews were blind to the previous screening results of the participants. The study took place in three rooms in a building close to the refugee accommodation.

### 2.5. Measures

#### 2.5.1. Refugee Health Screener

The RHS-15 (Hollifield et al., 2013) is a 15-item instrument screening for emotional distress in refugees. It was developed by selecting the most significant items from several diagnostic measures using a statistical multiple method approach (Hollifield et al., 2013). For previous studies, see the description in the introduction.

The RHS-15 is organized as follows: the first 13 items (RHS-13) comprise symptoms relating to the spectrum of depression, anxiety, and PTSD. The items are rated for the last month on a 5-point Likert scale (0 = *not at all* to 4 = *extremely*), visualized through pictures of bottles. Additionally, there is one coping item assessing the general ability to handle stress on a 5-point Likert scale and a distress thermometer (DT) for the last week ranging from 0 to 10. For a positive screening result, Hollifield et al. (2013) recommend a sum score of items 1 to 14 ≥ 12 and/or a DT ≥ 5. For the RHS-13, a total score ≥ 11 is recommended (Hollifield et al., 2016).

A translation of the questionnaire was already available for most of the native languages of the participants (Arabic, Farsi, Russian, Somali); translations into Albanian, Kurdish, and Serbian had to be obtained. To ensure a valid and precise translation, a written translation was generated, followed by a blind back-translation. Adequacy and differences between the translations were intensively discussed to guarantee an accurate translation.

The following versions of the RHS were examined in detail: (1) RHS-15 case (Items 1–14 ≥ 12 and/or DT ≥ 5), (2) RHS-13 case (Items 1–13 ≥ 11), (3) RHS-15 score (∑_z-transformed items 1–15_), (4) RHS-14 score (∑_items 1–14_), and (5) RHS-13 score (∑_items 1–13_).

#### 2.5.2. Self-rated screening

The self-rated screening consisted of the RHS-15 described above. Additionally, we included some sociodemographic questions as well as one feedback question about how difficult the refugees found the task of filling in the RHS-15 themselves on a 5-point Likert scale. A psychologist present during screening filled in an observational questionnaire about the amount of support needed by each person and the understanding of the questions.

#### 2.5.3. Semi-structured clinical interview

The first part of the interview consisted of the RHS-15 administered as an interview (for more details, see above) and sociodemographic questions. Hereinafter, the instruments used for validating the RHS are presented.

The Brief Symptom Inventory-18 (BSI-18; Derogatis, 2000) assesses depression, anxiety, and somatization symptoms as well as a global score for psychological distress. It shows good psychometric properties for adults and adolescents and has been used in various countries (e.g. Asner-Self, Schreiber, & Marotta, 2006; Franke et al., 2017). It consists of 18 items, six for each scale. The symptoms are rated on a 5-point Likert scale (0 = *not at all* to 4 = *extremely*). Following a previous study (Zabora et al., 2001), we used a cutoff score ≥ 10 for male and ≥ 13 for female as an indication for psychological distress. The BSI revealed good internal consistency in this sample (α = .93). For calculations, we used the sum score and the cutoff score. Additionally, Module C of the Mini International Neuropsychiatric Interview (MINI, version 5.0.0; Sheehan et al., 1998) was used to assess suicidality in the past month.

Daily functioning was assessed with eight self-constructed items rated on a 4-point Likert scale (0 = *not at all* to 3 = *severe*) selected because of their relevance for refugees (see Supplemental data File 2). Cronbach’s α for the present sample was .79. Calculations were performed with the sum score.

The Posttraumatic Stress Disorder Checklist-5 (PCL-5; Weathers et al., 2013b) was used to assess PTSD symptom severity and PTSD diagnosis according to DSM-5. Studies showed a good validity and reliability of the PCL-5 (e.g. Blevins, Weathers, Davis, Witte, & Domino, 2015). A recent study also showed that the PCL-5 is applicable for adolescents (Yang et al., 2017). The PCL-5 consists of 20 items rated on a 5-point Likert scale (0 = *not at all* to 4 = *extremely*). It is designed as a self-rating instrument; however, in this study, it was administered as a semi-structured clinical interview, rating both the severity and the frequency of the symptoms. To diagnose PTSD, we additionally assessed PTSD criteria F – G according to the DSM-5 criteria. As no definitions for sub-syndromal PTSD for DSM-5 are available to date, we defined it the following way: Criterion A and 2 or 3 of the criteria B – E have to be fulfilled. Cronbach’s α was .91 for this study. The sum score and PTSD diagnosis were used for calculations.

To assess trauma exposure, the Life Events Checklist (LEC-5; Weathers et al., 2013a) was used. Gray, Litz, Hsu, and Lombardo (2004) showed that the LEC has good psychometric properties. The questionnaire lists 17 categories of traumatic events. For calculations, we used the sum score of the different types of traumatic experiences that were personally experienced or witnessed.

To assess the refugees’ acceptance of the RHS-15 as a self-rating instrument, two questions rated on a 5-point Likert scale were formulated (*How would you rate your experience filling in the questionnaire?* and *Do you think that such a screening could be helpful for refugees?*).

### 2.6. Data analysis

All analyses were carried out with SPSS, version 23. Independent *t*-tests and Pearson correlations were computed for variables meeting the preconditions of parametric analyses. Mann-Whitney U and Spearman correlations were used for variables deviating from the preconditions (West, Finch, & Curran, 1995). Categorical and dichotomous variables were calculated with the likelihood ratio χ^2^, Fisher’s exact test, and McNemar. Correlations and phi were classified with .10 as a small, .30 as a moderate, and .50 as a large effect. We used an alpha level of 5% in all calculations. Missing values accounting for <10% of a scale were set as 0, participants with missing values >10% of a scale were excluded from the corresponding analyses. One outlier was detected in the RHS-15 self-rating; however, we did not exclude this outlier owing to content-related considerations.

Principal axis factoring analyses were only conducted for the RHS-15 and RHS-13 self-rating versions because the subject-to-variable ratio of the RHS self-rating (5.73:1) is adequate, however, it is deficient for the RHS interview version (3.73:1; Arrindell & Van Der Ende, 1985). We used Kaiser’s eigenvalue (EV) criterion (>1), the scree plot, and parallel analysis (Patil, Singh, Mishra, & Donavan, 2007) to determine the factor structure. To assess the homogeneity of the RHS, item-total and inter-item correlations were calculated. Item-total correlations above .20 are seen as appropriate (Streiner, Norman, & Cairney, 2015). Inter-item correlations between .2 and .4 are seen as an optimal level of homogeneity, values above .5 indicate the redundancy of some items because of equality (Briggs & Cheek, 1986).

To assess the reproducibility of the RHS self-rating and interview version, both agreement and reliability were measured. Agreement was measured with the smallest real difference (*SRD*) following De Vet, Bouter, Bezemer, and Beurskens (2001). Change scores of the participants between the RHS interview and self-rating were compared with the *SRD*. Higher or lower scores than the *SRD* were interpreted as ‘real’ change, i.e. above measurement error. The reliability was calculated with intraclass correlation coefficients (ICC) estimates based on a mean-rating, absolute-agreement, two-way random effects model (Koo & Li, 2016; McGraw & Wong, 1996). ICC estimates were interpreted as <.5 poor, .5–.75 moderate, .75–.90 good, and >.90 excellent reliability (Koo & Li, 2016).

To assess the predictive validity of the RHS, sensitivity, specificity, negative predictive value (NPV), positive predictive value (PPV), and Fisher’s exact tests were calculated. Positive cases were defined as participants with a PTSD diagnosis based on the PCL-5 or a symptom score above the cutoff of the BSI-18. The area under the receiver operating characteristics (ROC) curve (AUC), a measure of responsiveness, was calculated for the different RHS versions (Hajian-Tilaki, 2013). According to Terwee et al. (2007), an AUC of at least .70 is adequate.

## 3. Results

### 3.1. RHS, mental health, and traumatic experiences

Table 1 summarizes the mental health and traumatic experiences of the studied sample. Of the whole sample, 52% had a positive screening result in the RHS-15 and 42% in the RHS-13. The RHS-15 detected significantly more positive cases compared to the RHS-13 (self-rating: McNemar χ^2^(1) = 7.11, *p* < .01, ⱷ = .29, *n* = 86; interview: χ^2^(1) = 5.14, *p* < .05, ⱷ = .30, *n* = 56). The higher number of positive cases was due to the DT. No differences in the amount of positive cases were found between the self-rating and interview version.

**Table 1.** RHS, mental health, and traumatic experiences.

	% PC	(*n*)	*M (SD)*
RHS-15 self-rating	52	(45)^c^	∑_1–14_:14.09 (12.51)/DT: 3.29 (3.13)
RHS-15 interview	54	(30)^b^	∑_1–14_:12.59 (12.24)/DT: 3.93 (2.97)
RHS-13 self-rating	42	(36)^c^	13.00 (12.11)
RHS-13 interview	41	(23)^b^	11.55 (11.92)
Psychological distress (BSI-18)	35	(19)^a^	10.75 (13.46)
Suicidal thoughts (MINI-C)	16	(9)^a^	
PTSD (PCL-5)	13	(7)^a^	9.85 (11.58)
Sub-syndromal PTSD	22	(12)^a^	
Exposure to traumatic events (LEC-5) ≥ 2	100	(56)^b^	9.79 (4.93)

% PC = percentage of positive cases (above the according cutoff/fulfilling the diagnosis), ^a^ *n* = 55, ^b^ *n* = 56, ^c^ *n* = 86, RHS = Refugee Health Screener, DT = distress thermometer, BSI-18 = Brief Symptom Inventory-18, MINI-C = MINI International Neuropsychiatric Interview, Module C Suicidality, PTSD = Posttraumatic stress disorder, PCL-5 = Posttraumatic Stress Disorder Checklist-5, LEC-5 = Life Events Checklist-5.

### 3.2. Feasibility of the RHS-15 as a self-rating instrument

The majority (72%, *n* = 62) of the participants reported no difficulties in filling in the RHS. Small difficulties were reported by 20% (*n* = 17), moderate difficulties by 6% (*n* = 5), and extreme difficulties by 2% (*n* = 2; *M* for the reported difficulties = .40, *SD *= .76, range 0–4, *n* = 86). External report from the psychologists’ perspective was comparable, with the majority showing no need for support or difficulties in understanding. The need for support was rated with *M* = 1.00 (*SD* = 1.23, range 0–4, *n* = 81; 0 = *no support needed* to 4 = *very high support needed*), with most support being needed by illiterates. Understanding of the questions was on average *M* = .59 (*SD* = .86, range 0–3, *n* = 81; 0 = *no difficulties* to 4 = *extreme difficulties*), whereby the most common problems were present in the understanding of questions 14 and 15 as well as in the comprehension of the general scaling.

Participants with a higher level of school education showed less need for support (*r* = -.47, *p* < .001, *n* = 81) and fewer difficulties in understanding the questionnaire (*r* = -.50, *p *< .001, *n* = 81). No relationship between the amount of required support or difficulties in understanding with age or gender was found. Participants who had been in Germany for a longer period of time showed less need for support (*r *= .36, *p* < .01, *n* = 53), however no significant differences in the understanding of questions were found.

### 3.3. Reliability

#### 3.3.1. Internal consistency, item-total, and inter-item correlations

The principal axis factoring analysis for the RHS-15 self-rating version showed two factors with EV > 1. The first factor (EV = 7.51) accounted for 50%, the second factor (EV = 1.24) for 8%. However, the scree plot, the parallel analysis, as well as a content-related analysis of the two factors revealed a one-factor structure. For the RHS-13 self-rating version, all criteria revealed a one-factor structure accounting for 54% of the variance (EV = 7.00).

The different versions of RHS showed excellent internal consistency with Cronbach’s α ranging between .91–.93. All item-total correlations were significant. Inter-item correlations were slightly high, but acceptable (see Supplemental data File 3).

#### 3.3.2. Reproducibility: comparison of self-rating and interview version

Agreement was measured with the smallest real differences, *SRD* = 13.38 for RHS-15 and *SRD* = 15.93 for RHS-13. Changes between the interview and self-rating version higher than the *SRD* were found in 5% (*n* = 3 of 55) in the RHS-15 and 5% (*n* = 3 of 56) in the RHS-13. Additionally, we calculated a McNemar test that showed that there are no significant differences in the positive RHS cases between self-rating and interview version (RHS-15: χ^2^(1) = .10, *p* = .75, *n* = 56; RHS-13: χ^2^(1) = .00, *p* = 1.00). Intraclass correlation coefficients (ICCs) indicated a good reliability for RHS-15 (ICC = .86, CI [.76–.92]) and RHS-13 (ICC = .85, CI [.75–.92]). At the item-level, moderate to good reliabilities (ICC range .56–.84) were found for most of the items; however, three items (5, 12, 14) showed a poor reliability.

### 3.4. Validity

#### 3.4.1. Face validity

Participants rated their experience with the RHS as very good (45%, *n* = 24), good (38%, *n* = 20), and moderate (17%, *n* = 9), with *M* = 3.28 (*SD* = .74, range 2–4, *n* = 53; 0 = *very bad* to 4 = *very good*). Most of the participants thought that a screening for refugees could be helpful (*M* = 3.59, *SD* = .75, range 0–4, *n* = 51; 0 = *not at all* to 4 = *extremely*): Sixty-nine percent (*n* = 35) rated it as extremely helpful, 25% (*n* = 13) as quite helpful, 4% (*n* = 2) as moderately helpful, and 2% (*n* = 1) as not at all helpful.

#### 3.4.2. Predictive validity

Table 2 shows the sensitivity, specificity, PPV, and NPV of the RHS versions for the cutoff recommended by Hollifield et al. (2016). All RHS versions, both self-rating and interview, do an excellent job of predicting cases of clinically relevant depression, anxiety, and somatization symptoms, and PTSD. The interview version shows slightly higher sensitivity, specificity, PPV, and NPV than the self-rating version. Comparing the 15-item and the 13-item versions, the RHS-13 showed a slightly higher specificity and PPV compared to the RHS-15. On the other hand, the RHS-15 showed a slightly higher sensitivity and NPV than the RHS-13. The RHS-13 and RHS-15 version predicted both PCL-5 and BSI-18 cases well; however, specificity and PPV for PTSD cases were low. The AUC of the ROC measuring the responsiveness of the RHS showed adequate results for all versions (range AUC .89–.98). A balanced ratio of high sensitivity and specificity (specificity > .8) was considered for locating the optimal cutoff. Thereby, a cutoff of 13 for the RHS-13 and a cutoff of 14 for the RHS-14 was determined. Differentiating between self-rating and interview version would result in slightly higher cutoff values in the interview version, for example, 14 and 15 for RHS-13 and RHS-14 (for an overview, see Supplemental data File 4).

**Table 2.** Predictive validity of RHS-13 and RHS-15 cases.

		Comparison RHS self-rating to clinical rating						Comparison RHS interview to clinical rating					
		BSI and/or PCL case		PCL case		BSI case		BSI and/or PCL case		PCL case		BSI case	
		positive	negative	positive	negative	positive	negative	positive	negative	positive	negative	positive	negative
RHS-13 case	positive	15	8	6	16	15	8	18	5	7	15	18	5
	negative	4	29	1	32	4	28	1	32	0	33	1	31
	Statistic	LR χ^2^ = 17.65, *p* < .001		*p* < .05^a^		LR χ^2^ = 17.07, *p* < .001		LR χ^2^ = 38.70, *p* < .001		*p* < .001^a^		LR χ^2^ = 37.92, *p* < .001	
	Effect size	ⱷ = .55		ⱷ = .36		ⱷ = .55		ⱷ = .78		ⱷ = .47		ⱷ = .78	
	PPV/NPV	.65/.88		.27/.97		.65/.88		.78/.97		.32/1.00		.78/.97	
	Sens/Spec	.79/.78		.86/.67		.79/.78		.95/.87		1.00/.69		.95/.86	
RHS-15 case	positive	17	11	6	21	17	11	19	11	7	22	19	11
	negative	2	26	1	27	2	25	0	26	0	26	0	25
	Statistic	LR χ^2^ = 19.81, *p* < .001		*p* = .051^a^		LR χ^2^ = 19.13, *p* < .001		LR χ^2^ = 32.31, *p* < .001		*p* < .05^a^		LR χ^2^ = 31.48, *p* < .001	
	Effect size	ⱷ = .57		ⱷ = .28		ⱷ = .56		ⱷ = .67		ⱷ = .36		ⱷ = .66	
	PPV/NPV	.61/.93		.22/.96		.61/.93		.63/1.00		.24/1.00		.63/1.00	
	Sens/Spec	.90/.70		.86/.56		.90/.69		1.00/.70		1.00/.54		1.00/.69	

RHS = Refugee Health Screener, BSI = Brief Symptom Inventory-18, PCL = Posttraumatic Stress Disorder Checklist-5, BSI and/or PCL case = BSI-18 or PCL-5 OR BSI-18 and PCL-5 are positive, PCL case = PTSD diagnosis (DSM-5) fulfilled, BSI case = above BSI-18 cutoff, RHS-13 case = above RHS-13 cutoff, RHS-15 case = above RHS-15 cutoff, PPV = positive predictive value, NPV = negative predictive value, Sens = sensitivity, Spec = specificity, LR χ^2^ = likelihood ratio χ^2^, ⱷ = effect size phi, ^a^ Fisher’s exact test.

#### 3.4.3. Convergent validity

**Figure 2.**
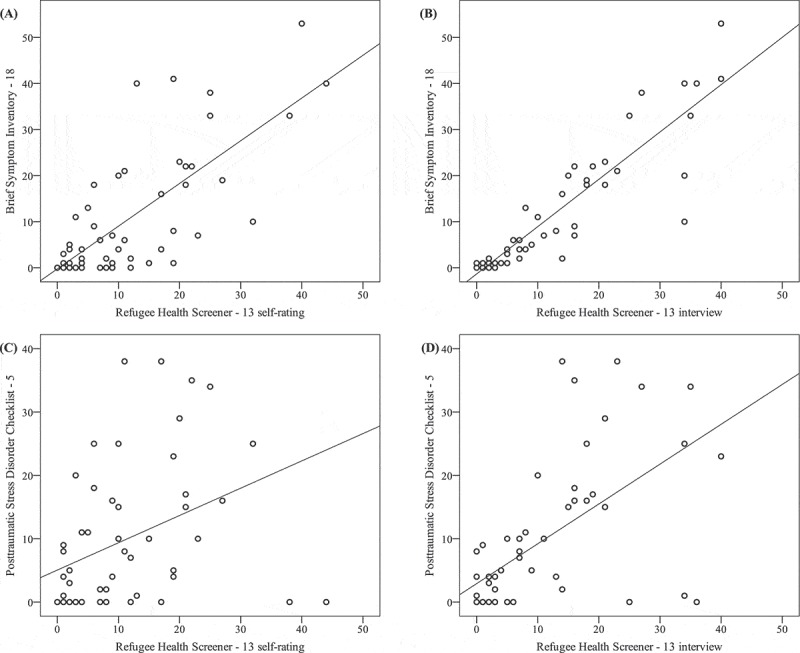
Correlations between the Brief Symptom Inventory-18 (BSI-18) and the Refugee Health Screener-13 (RHS-13) self-rating (A) and interview version (B), and between the Posttraumatic Stress Disorder Checklist-5 (PCL-5) and the RHS-13 self-rating (C) and interview version (D).

See Table 3 and Figure 2 for an overview of the correlations between the RHS and other mental health measures. All RHS versions are highly correlated with depression, anxiety, and somatization symptoms (see Table 3). This relation held when controlling for PTSD symptoms (range partial r (*pr)* = .63–.84, *p* < .001). The RHS versions also correlated with PTSD (see Table 3). However, when controlling for the BSI-18, correlations with the PTSD symptoms were only significant for the interview version (RHS-15: *pr* = .37, *p* < .05; RHS-13: *pr* = .30, *p* < .01). In general, correlations were higher for the interview version than for the self-rating version.

**Table 3.** Correlations between RHS and other mental health measures.

	RHS-13 self-rating	RHS-15 self-rating	RHS-13 interview	RHS-15 interview
BSI-18	.74*** ^b^	.77*** ^c^	.91*** ^b^	.90*** ^b^
Depression	.65*** ^b^	.69*** ^c^	.86*** ^b^	.87*** ^b^
Anxiety	.69*** ^b^	.72*** ^c^	.85*** ^b^	.84*** ^b^
Somatization^1^	.57*** ^b^	.59*** ^c^	.72*** ^b^	.72*** ^b^
Functioning	.52*** ^a^	.58*** ^b^	.62*** ^a^	.64*** ^a^
PCL-5	.37** ^b^	.42*** ^c^	.62*** ^b^	.65*** ^b^
LEC-5	.36** ^a^	.38** ^b^	.39** ^a^	.41** ^a^

^a^ *n* = 56, ^b^ *n* = 55, ^c^ *n* = 54, **p < .01, ***p < .001, ^1^Spearman rank order correlation, RHS = Refugee Health Screener, BSI-18 = Brief Symptom Inventory-18, PCL-5 = Posttraumatic Stress Disorder Checklist-5, LEC-5 = Life Events Checklist-5, Functioning = daily functioning.

## 4. Discussion

Despite EU regulations mandating the identification and support of vulnerable individuals, there are no adequately validated screening instruments available to detect the full range of mental health problems of the current refugee populations in Europe. This study examined the feasibility and validity of a candidate screening instrument, the Refugee Health Screener, in a refugee sample in Germany. The results are promising: The RHS successfully predicted clinically relevant symptoms of PTSD, depression, anxiety, or somatization, thereby detecting the most common mental health problems in refugees (Steel et al., 2009). Consistent with research conducted on refugee samples in the US (Hollifield et al., 2016, 2013; Johnson-Agbakwu et al., 2014; Polcher & Calloway, 2016), the RHS showed an excellent feasibility, validity, and reliability in the examined sample. It was not only applicable as an interview, but also showed good psychometric properties when carried out as a self-rating instrument, thus making it accessible for a larger variety of settings. The RHS-13, a shorter version consisting of the first 13 items, is even more time-efficient and equally valid.

In contrast to most other studies (e.g. Hollifield et al., 2016; Söndergaard et al., 2003), we did not focus on specific nationalities, but included all refugees with the aim of providing a real-world study. Notwithstanding the heterogeneity of the sample, the psychometric properties of the RHS were excellent, with a high predictability in the detection of mental health problems. The positive screening rates of previous RHS studies showed figures ranging from 23 to 46% (Hollifield et al., 2013, 2016; Johnson-Agbakwu et al., 2014; Polcher & Calloway, 2016). The higher screening rate of 52% in the RHS-15 self-rating version and 35% for clinically relevant depression, anxiety, and somatization symptoms in this study are comparable to prevalence rates of studies with similar refugee populations such as Führer et al. (2016; 55% depression, 40% anxiety disorder, 18% PTSD). However, the PTSD rate of 13% found in the semi-structured clinical interviews is comparatively low (Alpak et al., 2015; Führer et al., 2016), with a high number of participants showing a sub-syndromal PTSD (22%). Since PTSD depends on the cumulative exposure to traumatic stressors (Kolassa et al., 2010; Neuner et al., 2004), the PTSD rate will vary with the severity and frequency of life-threatening experiences. Further, manifest PTSD symptoms in refugees often appear after some time, when the so-called ‘honeymoon phase’ of euphoria and relief has passed (e.g. Sachs, Rosenfeld, Lhewa, Rasmussen, & Keller, 2008), but it can be detected quite early using the predictive utility of early depression and anxiety symptoms (Smid, Lensvelt-Mulders, Knipscheer, Gersons, & Kleber, 2011).

The one-factor solution of the RHS found in our study and in Hollifield et al. (2016) underlines that the RHS does not seem to specify between different disorders. The non-significant correlation between the RHS and PTSD symptoms when controlling for depression, anxiety, and somatization symptoms is therefore not surprising. This might be due to the high comorbidity of PTSD with clinically relevant depression, anxiety, and somatization symptoms in the present sample.

The interview and self-rating version of the RHS showed a good reproducibility, thereby indicating that the RHS can be administered both as a self-rating and as an interview. However, we found items 5, 12, and 14 to have a low reliability. Possible reasons for this could be notable symptom fluctuations across time or a bias, for instance, in terms of social desirability. In addition to the good reproducibility, the majority of refugees were able to fill in the RHS on their own, and the self-rating version only showed a slightly lower predictive value for mental health problems. Furthermore, the participants’ acceptance of the self-rated screening was very high, with a denial rate of 3%, and the vast majority of participants reporting the screening to be a good experience and a helpful tool for refugees. This goes hand in hand with other studies reporting similarly high acceptance rates (e.g. Hollifield et al., 2016). These findings are especially important, as a self-rating instrument could be more easily integrated into an initial medical screening or be used by people working with refugees, such as social workers or teachers. Similarly, Söndergaard et al. (2003) showed that a mental health screening can be administered by laypersons. Nevertheless, we recommend that an interpreter is present in case of a positive screening or if problems in filling in the questionnaire arise. Furthermore, illiteracy and education level should be considered when deciding upon the mode of administration. People using the RHS should be trained in culturally sensitive ways of introducing a mental health screening, offering psychoeducation to those with a positive screening result and, if desired, referring the refugees to mental health institutions. When using the RHS or any other screening, it is essential to provide a confidential setting, to use general health vocabulary, and to avoid potentially stigmatizing meanings of terms connected with mental illnesses (Al-Obaidi et al., 2015; Hollifield et al., 2013).

The present study showed possible future advancements for use in practice and research: The shorter 13-item version of the RHS introduced by Hollifield et al. (2016) revealed to be more time-efficient and feasible, with a gain in specificity and only a minor loss in sensitivity. The two excluded items were most often the reason for understanding problems and, accordingly, occupied more time. Furthermore, despite the good ability to detect clinically relevant symptoms of PTSD, depression, anxiety, and somatization, the RHS may overestimate the intensity of symptoms in some refugees. Two contributing factors were the DT and the cutoff score recommended by Hollifield et al. (2013). The DT led to an increase in positive cases of approximately 10%, thereby detecting any kind of stress, partially unconnected to mental health problems. The cutoff recommended by Hollifield et al. (2016) could be slightly adjusted with a cutoff of 13 for the RHS-13 and a cutoff of 14 for the RHS-15. In general, the choice for a specific cutoff should also reflect the subsequent options for further diagnosis and treatment. It seems plausible to first offer immediate diagnostic procedures to those with the highest scores.

Consequently, the validation of the RHS for refugees in Europe suggests that it is both suitable for use in further studies and as a practical tool for screening in existing health systems. Based on experiences of large-scale programmes in the US (e.g. Hollifield et al., 2016; Savin et al., 2005), we recommend the inclusion of a mental health screening such as the RHS in the initial medical examination. Refugees with a positive screening result should be offered psychoeducation and if the person wishes so, a referral to mental health services should be organized. Consequences of the implementation of such a mental health plan for health systems and refugees have not been studied systematically. Accordingly, future research should include large-scale studies implementing an all-embracing mental health plan for refugees (e.g. Elbert et al., 2016), thereby specifying the validity and reliability of the RHS for refugees with different nationalities, referral rates, the use of mental health services, and its consequences on refugees’ mental health and the health care system.

Certain limitations should be considered while interpreting the findings of the study: The sample size of the various nationalities and accordingly spoken languages did not allow for a meaningful differentiation in accounting for the various cultural backgrounds. Despite the potential respective variations, the correlations between the self-rating and interview versions were high. Assessing the RHS self-rating and interview versions in the same order for all participants led to different degrees of familiarity. Furthermore, the time period between the self-rated screening and the interview could have led to symptom changes between the assessments. However, this would have reduced rather than produced significant correlations between the measures. The BSI-18 indicates symptoms of depression, anxiety, and somatization, but certainly does not allow for a respective diagnosis. Both the self-rating and the interviews rely on the subjective reports of the participants. Potential bias, such as social desirability or assumptions about a potential connection to the asylum procedure, may have added noise or biases despite intense explanation of the study aims to each participant.

## 5. Conclusion

The present study tackles the lack of valid mental health screening instruments for current refugees in Europe. The Refugee Health Screener, which has already shown to be a valid screening instrument in refugee populations in the US, also shows excellent feasibility, validity, and reliability in the examined refugee sample in Germany. It detects clinically relevant mental health problems such as PTSD, depression, anxiety, and somatization symptoms, and is feasible and valid both as a self-rating questionnaire and as an interview. The shorter 13-item version is even more time-efficient and equally valid. As refugees frequently present with serious mental health problems that prevent integration into the host community, we suggest the inclusion of a mental health screening in the initial medical examination. In the case of a positive screening result, referral to mental health services for in-depth diagnostic and treatment is mandatory. Because of its excellent psychometric properties, its simple feasibility, and the high acceptance of even vulnerable refugees, we recommend the RHS as such a screening instrument. However, the capacities for supporting and treating a large number of refugees in need of mental health care within the host countries also need to be established, as suggested, for example, by Elbert et al. (2016). Based on existing evidence, we hold that early detection and evidence-based treatment of mental health problems is imperative both on humanitarian grounds and as a cost-effective measure, as it can substantially improve psychosocial functioning and enhance integration (Bozorgmehr & Razum, 2015; Lamkaddem et al., 2014; Schick et al., 2016; Song et al., 2015).

## Supplementary Material

Supplementary material
