# Supplementary material for: Efficient identification of mental health problems in refugees in Germany: the Refugee Health Screener
Source: Eur J Psychotraumatol. 2017 Nov 7;8(sup2):1389205. doi: 10.1080/20008198.2017.1389205 (PMC5687797; doi:10.1080/20008198.2017.1389205)
Supplement: Supplementary material [file ZEPT_A_1389205_SM5620.docx]

Supplementary File 1. Sociodemographic characteristics and group differences

| Characteristic | | Total sample  (N = 86) | Screening only  (*n* = 30) | Interview  (*n* =56) | Statistic | *p* value |
| --- | --- | --- | --- | --- | --- | --- |
| Female sex, No. (%) | | 31 (36) | 11 (37) | 20 (36) | LR χ^2^=.01 ^c^ | 1 |
| Age, *M* (*SD*, range), y | | 28.76 (11.23, 12.08 –65.83) | 28.62 (9.09, 17.17 – 47) | 28.84 (12.32, 12.08-65.83) | t(83)=-.09 ^b^ | .933 |
| Education, *M* (*SD*, range), y | | 10.44 (4.01, 0 – 20) | 10.48 (4.82, 0 – 20) | 10.41 (3.57, 0 – 17) | t(83)=.08 ^b^ | .938 |
| Country of origin, No. (%) | |  |  |  | LR χ^2^=13.98 ^c^ | .159 |
| Syria, No. (%) | 50 (58) | 14 (47) | 36 (64) | LR χ^2^=2.48 | .168 |  |
| Afghanistan, No. (%) | 8 (9) | 2 (7) | 6 (11) | ^a^ | .708 |  |
| Albania, No. (%) | 7 (8) | 3 (10) | 4 (7) | ^a^ | .691 |  |
| Kosovo, No. (%) | 6 (7) | 3 (10) | 3 (5) | ^a^ | .416 |  |
| Serbia, No. (%) | 6 (7) | 3 (10) | 3 (5) | ^a^ | .416 |  |
| Iraq, No. (%) | 3 (4) | 0 (0) | 3 (5) | ^a^ | .549 |  |
| Macedonia, No. (%) | 2 (2) | 1 (3) | 1 (2) | ^a^ | 1 |  |
| Somalia, No. (%) | 2 (2) | 2 (7) | 0 (0) | ^a^ | .119 |  |
| Georgia, No. (%) | 2 (2) | 2 (7) | 0 (0) | ^a^ | .119 |  |
| Stay in Germany, *M* (*SD*, range), months | | 6.53 (2.99, 3 – 24) | 7.43 (4.49, 3 – 24) | 6.05 (1.59, 3 – 10) | U=735.5, z=-.97 ^b^ | .333 |
| RHS-13 self-rating score, *M* (*SD*, range) | | 13.00 (12.11, 0 – 50) | 15.5 (14.27, 0 – 50) | 11.66 (10.68, 0 – 44) | U=719.5, z=-1.09 ^c^ | .274 |
| RHS-14 self-rating score, *M* (*SD*, range) | | 14.09 (12.51, 0 – 53) | 16.77 (14.65, 0 – 53) | 12.66 (11.08, 0 – 46) | t(84)=1.46 ^c^ | .148 |
| RHS-15 case, No. (%) | | 45 (52) | 17 (57) | 28 (50) | LR χ^2^=.35 ^c^ | .652 |
| RHS-13 case, No. (%) | | 36 (42) | 13 (43) | 23 (41) | LR χ^2^=.04 ^c^ | 1 |

*Notes.* y = years, LR χ^2^= likelihood ratio χ^2^, ^a^ Fisher’s Exact Test, ^b^ *n* = 85, ^c^ *n* = 86, RHS = Refugee Health Screener.

Supplementary File 2. Self-constructed questionnaire to assess daily functioning

| Have you been impaired in your daily life by mental problems in the last 4 weeks? | Not at all | A little bit | Quite a bit | Severe | Not applicable |
| --- | --- | --- | --- | --- | --- |
| 1. Relationships to family members |  |  |  |  |  |
| 1. Relationships to friends |  |  |  |  |  |
| 1. Household chores and duties |  |  |  |  |  |
| 1. Fun and leisure activities |  |  |  |  |  |
| 1. Work |  |  |  |  |  |
| 1. School / education / academic studies |  |  |  |  |  |
| 1. General satisfaction with life |  |  |  |  |  |
| 1. Overall functioning in all areas of life |  |  |  |  |  |

Supplementary File 3. Cronbach’s α, item-total, and inter-item correlations

|  | RHS-13 self-rating | | RHS-15 self-rating | | RHS-13 interview | | RHS-15 interview | |
| --- | --- | --- | --- | --- | --- | --- | --- | --- |
|  | M | range | M | range | M | range | M | range |
| Cronbach’s α | .93 ^a^ |  | .91 ^b^ |  | .91 ^c^ |  | .91 ^c^ |  |
| Inter-item correlations | .49 ^a^ | .26-.75 | .45 ^b^ | .13-.75 | .45 ^c^ | -.06-.71 | .42 ^c^ | -.06-.74 |
| Item-total correlations | .68 ^a^ | .53-.85 | .64 ^b^ | .41-.83 | .64 ^c^ | .30-.81 | .62 ^c^ | .30-.84 |

*Notes.* ^a^ *n* = 86, ^b^ n = 84, ^c^ n = 56, RHS = Refugee Health Screener.

Supplementary File 4. Sensitivity, specificity, and AUC of the RHS

|  |  | RHS score | | | | | | | | | | | |  |  |  |
| --- | --- | --- | --- | --- | --- | --- | --- | --- | --- | --- | --- | --- | --- | --- | --- | --- |
| BSI-18 / PCL-5 case |  | 8 | 9 | 10 | 11 | 12 | 13 | 14 | 15 | 16 | 17 | 18 | 19 | AUC | 95% CI | *p* |
| RHS-13 Self-rating | Sensitivity | .90 | .90 | .90 | .79 | .74 | .74 | .68 | .68 | .68 | .68 | .63 | .63 | .89 | [.81, .98] | < .001 |
|  | Specificity | .62 | .68 | .76 | .78 | .81 | .87 | .87 | .87 | .89 | .89 | .92 | .92 |  |  |  |
| RHS-13 Interview | Sensitivity | 1.00 | .95 | .95 | .95 | .95 | .95 | .95 | .90 | .84 | .79 | .79 | .68 | .98 | [.95, 1.00] | < .001 |
|  | Specificity | .78 | .81 | .84 | .87 | .89 | .89 | .92 | .95 | .95 | 1.00 | 1.00 | 1.00 |  |  |  |
| RHS-14 Self-rating | Sensitivity | .90 | .90 | .90 | .90 | .84 | .79 | .74 | .68 | .68 | .68 | .68 | .68 | .90 | [.82, .98] | < .001 |
|  | Specificity | .62 | .62 | .65 | .76 | .78 | .81 | .84 | .87 | .87 | .87 | .89 | .95 |  |  |  |
| RHS-14 Interview | Sensitivity | 1.00 | 1.00 | .95 | .95 | .95 | .95 | .95 | .90 | .90 | .90 | .84 | .84 | .98 | [.95, 1.00] | < .001 |
|  | Specificity | .76 | .78 | .81 | .81 | .87 | .87 | .89 | .92 | .95 | .95 | .97 | .97 |  |  |  |

*Notes.* AUC = Area under the curve, CI = confidence interval, RHS = Refugee Health Screener, BSI-18 = Brief Symptom Inventory – 18, PCL-5 = Posttraumatic Stress Disorder Checklist – 5.
